# Metabolomics Differences of *Glycine max* QTLs Resistant to Soybean Looper

**DOI:** 10.3390/metabo11100710

**Published:** 2021-10-19

**Authors:** Maryam Yousefi-Taemeh, Jie Lin, Demian R. Ifa, Wayne Parrott, Nik Kovinich

**Affiliations:** 1 Department of Chemistry, York University, Toronto, ON M3J 1P3, Canada; yousefi@my.yorku.ca (M.Y.-T.); ifadr@yorku.ca (D.R.I.); 2 Department of Biology, York University, Toronto, ON M3J 1P3, Canada; lj0215@yorku.ca; 3 Center for Applied Genetic Technologies, University of Georgia, Athens, GA 30602, USA; wparrott@uga.edu

**Keywords:** *Glycine max*, insect resistance QTLs, *Chrysodeixis includens*, mass spectrometry, metabolomics, isoflavonoids

## Abstract

Quantitative trait loci (QTLs) E and M are major soybean alleles that confer resistance to leaf-chewing insects, and are particularly effective in combination. Flavonoids and/or isoflavonoids are classes of plant secondary metabolites that previous studies agree are the causative agents of resistance of these QTLs. However, all previous studies have compared soybean genotypes that are of dissimilar genetic backgrounds, leaving it questionable what metabolites are a result of the QTL rather than the genetic background. Here, we conducted a non-targeted mass spectrometry approach without liquid chromatography to identify differences in metabolite levels among QTLs E, M, and both (EM) that were introgressed into the background of the susceptible variety Benning. Our results found that E and M mainly confer low-level, global differences in distinct sets of metabolites. The isoflavonoid daidzein was the only metabolite that demonstrated major increases, specifically in insect-treated M and EM. Interestingly, M confers increased daidzein levels in response to insect, whereas E restores M’s depleted daidzein levels in the absence of insect. Since daidzein levels do not parallel levels of resistance, our data suggest a novel mechanism that the QTLs confer resistance to insects by mediating changes in hundreds of metabolites, which would be difficult for the insect to evolve tolerance. Collective global metabolite differences conferred by E and M might explain the increased resistance of EM.

## 1. Introduction

The use of crop protectants and fertilizers has helped maintain crop productivity, but these have also masked the fact that yield loss from insects continues to increase [1]. In the case of soybean, freshwater ecotoxicity has tripled, largely due to increased insecticide application, particularly ones that are more persistent in the soil and water [2]. Efforts to lower the cost of production, along with increased concerns over insecticide residues in the food chain and environment, underscore the need for insect-resistant crops [3].

Soybean is one of the crops that is affected by defoliating insects, particularly in the Southeastern United States and in tropical South America. The use of soybean engineered with Bt (*Bacillus thuringiensis*) is a success story for plant resistance to insects. However, Bt is not deployable in all areas and crops [4]. Case in point, soybean is a refuge for Bt corn and cotton in the United States, so Bt soybean cannot be commercialized in the USA without changes to Bt corn and cotton resistance management strategies. Thus, other sources of resistance are needed. Even in South America where Bt soybean is used [5], pyramiding other resistance genes with Bt is needed to obtain a more durable resistance.

Other resistance genes are available in the form of quantitative trait loci (QTLs) that confer resistance to defoliating insects in soybean [6]. Yet, almost nothing is known about the biochemical basis for such QTL-based resistance in soybean, or in most other crops. The first QTL was found on Linkage Group (LG) M (now chromosome 7) of a landrace, “Sodendaizu” (PI 229358) [7,8]. Since then, breeders have introgressed QTL-M (referred to henceforth as M) into diverse genetic backgrounds, showing its effectiveness is not genotype-dependent [9,10]. Other groups have also mapped resistance to the same chromosomal region [11,12].

The second important QTL comes from LG E found in the landrace “Miyako White” (PI 227687), and is hence known as QTL-E (or E) [8]. E is the same QTL initially identified by Terry et al. [13]. To better study these QTLs and their interactions, they were bred into a set of near-isolines [14]. When tested under laboratory and field conditions, the near isoline containing both M and E shows agriculturally effective levels of resistance to a gamut of defoliating caterpillars [15]. Based on field trials, soybean without a resistance QTL reaches the economic threshold of 35% defoliation by eight days after caterpillars, which is when insecticide use would be warranted. This timeline also holds for the isoline carrying E by itself. The line carrying only M does not reach the economic threshold until the 10th day, while the line with both E and M takes 12.5 days to reach the threshold [15]. Nevertheless, despite 40 years of study, the chemical nature of this defoliator resistance remains an enigma.

Many of the original biochemical characterizations were done on soybean carrying E, with the resistance being attributed to various compounds at various times. Smith and Fischer determined that resistance was due to methanol-soluble compounds [16]. Glyceollins are isoflavonoid-derived phytoalexins that have well-established roles in protecting soybean from microbial pathogens [17,18,19]. Yet, Hart et al. ruled out glyceollins as a source of resistance to leaf-chewing insects [20]. Caballero & Smith attributed resistance to coumestrol, phaseol, and afrormosin [21]. Sharma and Norris showed that a combination of daidzen, glyceollins, sojagol, and coumestrol, along with an unidentified compound, was involved in resistance [22]. In contrast, Liu et al. showed glyceollins, and not coumestrol, led to resistance [23,24]. Alternatively, Piubelli et al. provided evidence that the resistance is due to rutin and genistin [25]. Hoffman-Campo et al. also implicated rutin in resistance [26].

All of these studies implicate isoflavonoids and/or flavonoids as being responsible for the resistance of E, even if the results are contradictory and inconclusive when it comes to which particular compound confers resistance. Altogether, different results were obtained from a study based on transcripts upregulated upon insect feeding rather than on metabolomics. Wang et al. identified vegetative storage protein β (*GmVSPb*), NADPH:isoflavone reductase (*GmN:IFR*), and allene oxide synthase (*GmAOS1*) as genes providing resistance, but these do not co-map with any of the reported QTLs [27,28], suggesting they are downstream in the response cascade.

The basis for M-mediated resistance is no clearer. Two genotypes containing M were found to both produce kaempferol 3-*O*-α-L-rhamnopyranosyl-(1→4)-[α-L-rhamnopyranosyl-(1→6)-β-D-galactopyranoside] and quercetin 3-*O*-β-D-glucopyranosyl-(1→2)-[α-L-rhamnopyranosyl-(1→6)-β-D-galactopyranoside]. In contrast, Zhao et al. associated the production of elevated genistein and glycitein with the increased resistance provided by M [29]. Recently, Gómez et al. found that compared with the control, a soybean cultivar with M had higher levels of the flavonoids kaempferol-3-*O*-L-rhamnopyranosyl-glucopyranoside, rutin (quercetin 3-*O*-rutinoside), quercetin-3,7-*O*-di-glucoside, quercetin-3-*O*-rhamnosylglycoside-7-*O*-glucoside, quercetin-3-*O*-rhamnopyranosyl-glucopyranoside-rhamnopyranoside, and the isoflavonoids genistein-7-*O*-diglucosidedimalonyl, genistein-7-*O*-6-*O*-malonylglucoside, and daidzein 7-*O*-glucoside-malonate [30]. In a follow-up study, Gómez et al. identified isorhamnetin glycoconjugates in the resistant genotype, along with increased levels of proteinase inhibitors and flavonoids [31].

All of these studies have been confounded by the use of different genotypic backgrounds for the QTLs being studied. Therefore, it has never been clear whether the presence of some compounds is simply due to the choice of genetic background, rather than being causative of resistance. Thus, the goal of this work was to characterize metabolome differences of near isogenic lines containing QTLs E, M, or both (EM) that were introgressed into the background of the susceptible variety Benning in an effort to identify differences that are specifically a result of these QTLs. The use of near isogenic lines minimizes the effects of different genetic backgrounds and increases the chance that the observed effects are due to the QTLs.

## 2. Results

### Mass Spectrometric Comparision of Metabolites from Soybean QTLs Resistant to Soybean Looper

Principal component analysis (PCA) and orthogonal PLS-PA were unable to distinguish the genotypes or treatments based on the 13,950 unique peaks that were measured (Supplementary Figure S1). However, the genotypes were found to have significant differences based on their collective peak intensities (ANOVA, *p* < 1.42 × 10^−11^). Each insect-treated genotype was significantly different from all others (Tukey’s post hoc, *p* < 0.01). Under mock treatment, all were significantly different except for QTL M compared to Benning.

**Figure 1:**
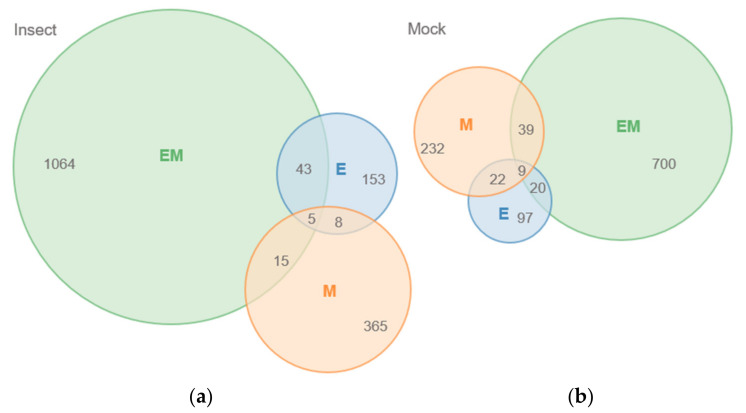
Venn diagrams of the number of MS peaks from soybean QTLs that are significantly different in intensity from insect-susceptible parent Benning. (**a**) Treatment with soybean looper for 72 h. (**b**) No insect treatment.

Of the 13,950 unique peaks, 1521 and 957 peaks were significantly different (up- or down-accumulated) in at least one genotype compared to Benning upon insect and mock treatments, respectively (paired *t*-test, *p* < 0.05). EM demonstrated the greatest number of differences, with 1064 and 700 peaks different from those of Benning under insect and mock treatments, respectively (Figure 1). M had the second greatest number of differences, with 365 and 232 under insect and mock treatments. EM and M shared 39 differences under mock treatment and 15 in response to insect. In contrast, the number of peaks shared by EM and E increased from 20 to 43 in response to the insect treatment. A list of shared peaks is provided in (Supplementary Table S1).

**Figure 2:**
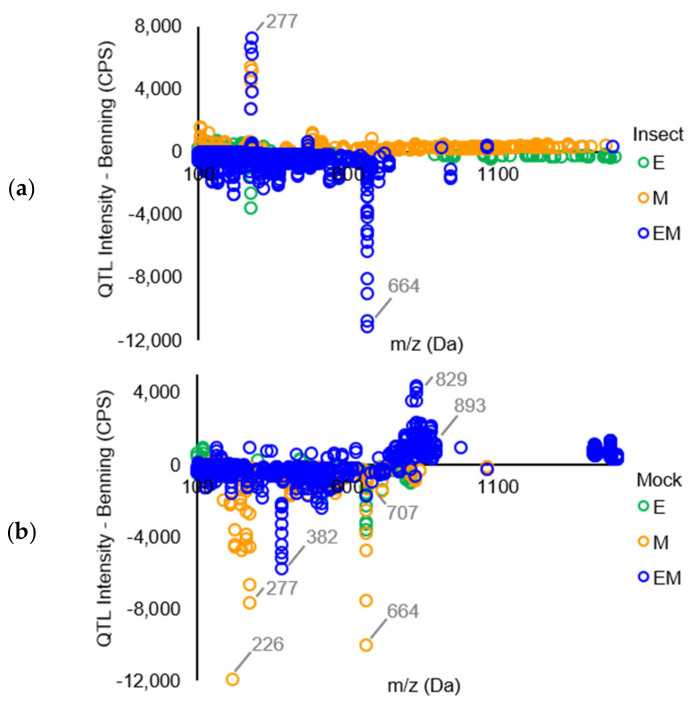
Comparison of average full scan mass spectra of soybean QTLs compared to Benning showing the increase or decrease of metabolites. Only mass spectra that were significantly different in intensity compared to Benning are shown. (**a**) Treatment with soybean looper for 72 h. (**b**) No insect treatment.

A plot of those significantly different peaks revealed that relatively few (only 4.3% and 10.2% from insect- and mock-treated QTLs, respectively) differed in intensity by more than 1500 CPS compared to the peaks from Benning (Figure 2). Under insect treatment, only peaks corresponding to the isoflavonoid daidzein [*m*/*z* 277, [M + Na]^+^] showed major increases compared to Benning (Figure 2a). This identity was confirmed by the spiking of samples with authentic standard. The elevated daidzein levels were found in EM and M, not in E. Under mock treatment, the most elevated peaks included an unknown at *m*/*z* 829, putative soyasaponin Ya (*m*/*z* 893), and soyasaponin Bd (*m*/*z* 978), which were most abundant in EM. Under mock treatment, most peaks that were shared by all three QTLs had reduced levels compared to those of Benning. These included the saponins ruscoponticoside C (*m*/*z* 707) and combreglucoside (*m*/*z* 664), an unknown compound at *m*/*z* 382, and the salicylate salicortin (*m*/*z* 226 [M + H − 2H2O]^+^). A subset of compounds that had major changes in intensity among the genotypes were selected for identification by MS/MS (Supplementary Table S2).

A graph of daidzein demonstrates that the levels decrease in Benning and E upon insect treatment. By contrast, levels are lower in mock M, but increase upon insect treatment. In EM, mock levels are similarly as high as Benning mock and do not decrease with insect treatment.

## 3. Discussion

The soybean QTLs E and M provide notable levels of resistance to leaf-chewing insects [8,15,32]. The amounts of flavonoids and isoflavones are known to increase in soybean leaves in response to defoliating insects [33]. While previous studies agree that flavonoid and/or isoflavonoid metabolites are the causative agents of resistance, studies have always been on soybean genotypes that are of dissimilar genetic backgrounds. Thus, it has never been clear whether the presence of particular metabolites has been due to genetic background or specifically to the QTLs that provide resistance. Here, we compared the metabolite profiles of QTLs E, M, and both (EM) that have been introgressed into the background of the susceptible genotype Benning. Our non-targeted MS analysis generally found global, relatively low-level changes in metabolite composition compared to Benning, whether the isolines were treated with insects or not (Figure 1 and Figure 2). Thus, resistance could be due to differences in the abundance of a wide variety of metabolites, rather than the overaccumulation of just one or a few compounds, as was previously suggested. In this context, the enhanced resistance of EM over individual QTLs could result from the distinct sets of metabolites contributed by E and M.

**Figure 3:**
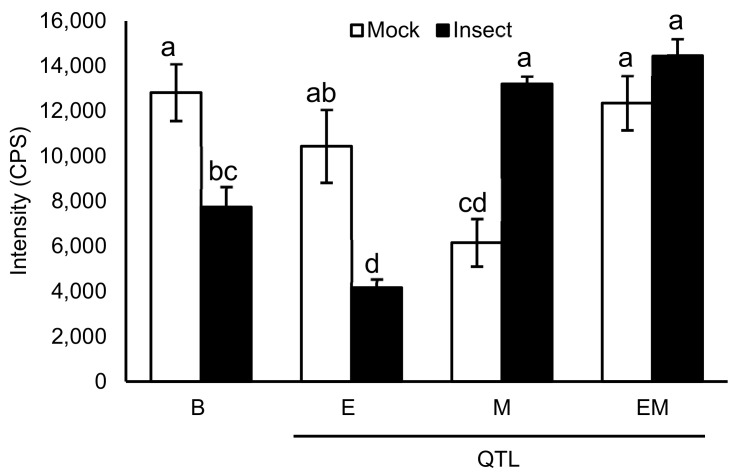
Average daidzein levels in mock and insect treated leaves of soybean QTLs and the insect-susceptible parent Benning. Different letters show significant differences by single factor ANOVA, Tukey post hoc test (*p* < 0.05, α = 0.05). Error bars indicate SE (*n* = 3 biological replicates).

Daidzein has been shown to be toxic to insects [34,35,36] and was previously associated with insect resistance [34,35,37]. Since daidzein is the only metabolite that demonstrates major increases in M and EM compared to Benning (Figure 2), we focused on it for a more in-depth analysis. Benning demonstrates relatively high levels of daidzein under mock treatment, yet levels exhibit major decreases upon insect treatment (Figure 3). The same was observed for E. Thus, in Benning and E, soybean looper may be able to suppress daidzein biosynthesis, enhance its degradation, and/or its conversion into glycosylated derivatives.

M has lower levels of daidzein compared to Benning under mock treatment. Yet, in contrast to Benning and E, daidzein levels increase upon insect treatment rather than decrease (Figure 3). This opposite response may suggest a signaling role for M, where M counteracts the suppressive mechanism of soybean looper seen in Benning and E.

**Figure 4:**
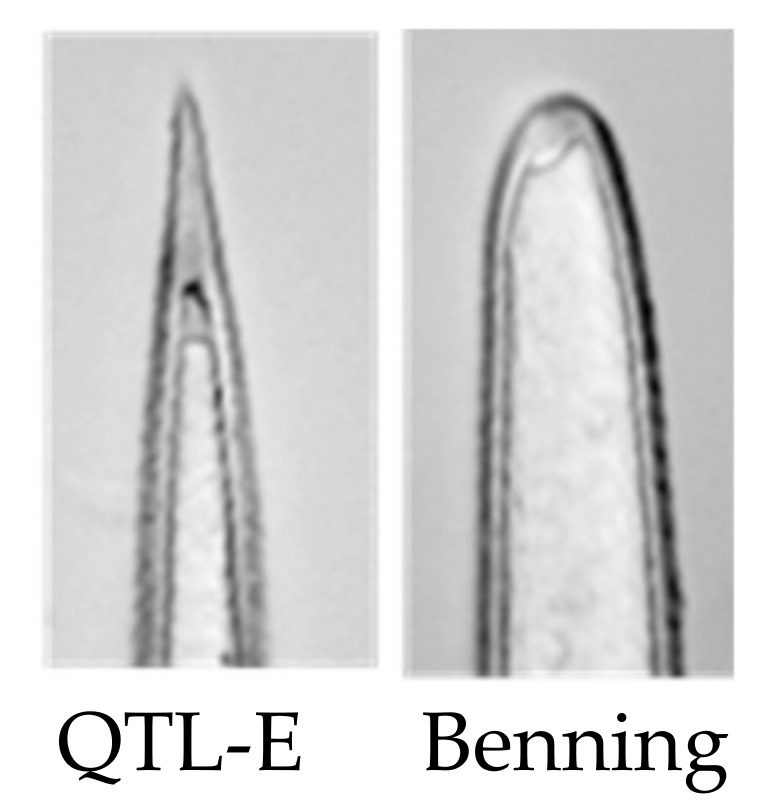
Trichomes of QTL E in the Benning background have sharp tip shape, whereas insect-susceptible Benning does not.

E has lower levels of daidzein under mock and insect treatments, suggesting that the mechanism of resistance is independent of daidzein. Consistent with this, soybean varieties Enrei and Tamahomare are susceptible genotypes that have high levels of diadzein [38,39]. E is a 2-Mb region on chromosome 15 that often co-segregates with the *Pb* locus for sharp-tipped trichomes (Figure 4) [40]. Although there are earlier reports on the effect of pubescence traits on soybean resistance to insect [41,42], Hulburt was the first to report that a sharp-trichome locus co-localizes with E [43]. Most soybean cultivars have blunt trichomes (*pb*) while the wild soybean, *Glycine soja*, almost universally has sharp trichomes [44]. Since some *G. soja* lines are more susceptible to defoliating insects than most *G. max* lines, it seems unlikely that the trichome tip alone governs resistance, despite the genomic collocation of the two traits [40]. Lambert and Kilen showed that PI 227687’s resistance is graft-transmissible, confirming that resistance in E is tissue-mobile metabolites or macromolecules [45].

## 4. Materials and Methods

### 4.1. Chemicals

Methanol (HPLC grade, ≥99.9% purity) and water (HPLC grade) were purchased from Sigma-Aldrich. (St. Louis, MO, USA). Daidzein was obtained from Indofine Chemical Company (Hillsborough, NJ, USA).

### 4.2. Plant Growth Conditions

Plants of Benning, Benning M, Benning E, and Benning EM [14] were grown until the V5 growth stage [46] at 27 °C in an insecticide-free greenhouse under 16 h:8 h light:dark to prevent premature flowering. Each plant was grown in a 0.95 L styrofoam cup with three drainage holes punched in the bottom and filled with Fafard 3B potting mix (Conrad Fafard, Agawam, MA, USA). The pots were fertilized with approximately 75 Osmocote^®^ 15-9-12 pellets (Scotts Miracle-Gro, Marysville, OH, USA) per cup at the V2 growth stage. At the V5 stage, three soybean looper neonate larvae were applied to each plant.

### 4.3. Insect Treatments

The experimental design was a randomized complete block design with ten blocks. Each block consisted of the four genotypes under two treatments: thirty neonate soybean looper, *Chrysodeixis includens*, larvae (Benzon Research Inc., Carlisle, PA, USA) were added to one treatment, while the control treatment received no insects. At the 72-h timepoint after infestation, the first fully expanded trifoliolate leaf was collected from each plant. Leaves were flash frozen in liquid nitrogen and stored at −80 °C. Frozen leaves were homogenized by crushing and subsamples of the crushed, frozen leaves were lyophilized and ground to a powder using a bead mill.

### 4.4. Extraction and Sample Preparations

Fresh and freeze-dried leaf extracts were prepared and normalized by adding 25 μL of extraction solution (methanol and 80% methanol, respectively) per mg of tissue sample. The mixtures were shaken overnight at 100 rpm and then centrifuged at 12,000× *g* for 30 min. The supernatants were transferred into new vials following another centrifugation at the speed of 12,000× *g* for 30 min. The sample mixtures were passed through 0.2 μm filter and the extracted samples were kept in Eppendorf vials for further analyses.

Prior to injections in the ESI-MS system, a (1:25) dilution step was performed in acidic solution (80% methanol containing 1% formic acid), or basic solution (80% methanol containing 1% ammonium hydroxide) for positive and negative ion mode analyses, respectively.

The extracts of fresh and freeze-dried samples were analyzed in both positive and negative electrospray ionization (ESI) modes.

As a result, the freeze-dried leaf extracts showed greater intensities for most peaks and was chosen over fresh samples for the next series of experiment.

Next, samples previously discussed in Section 4.2 and Section 4.3 were provided in biological triplicate (4 genotypes × 2 treatments × 3 replicates). For the positive and the negative ion mode analyses, samples were diluted to a 1:10 ratio, in acidic and basic solutions as explained before.

### 4.5. Metabolite Analysis by ESI-MS

Direct infusion of diluted samples was performed in an LTQ linear ion trap mass spectrometer (Thermo Fisher Scientific, San Jose, CA, USA), which is controlled by Xcalibur 2.0 software and is equipped with an ESI ion source.

The parameters for ESI-MS analysis were chosen as follows: Nitrogen at 100 psi was used as the nebulizing gas for all the experiments. The flow rate, maximum ion trap injection time, and microscans were 10 μL min^−1^, 20 ms, and 3 microscans per spectrum, respectively. The analyses were performed in full scan mode, in the range of *m*/*z* 150 to 1500. For the negative ion mode analyses, the ESI source parameters were as follows: spray voltage: −5 kV; capillary temperature: 250 °C, capillary voltage: −10 V, tube lens: −150 V, and sheath gas flow rate: 30. For the positive ion polarity analyses, the ESI source parameters were as follows: spray voltage: 5.5 kV; capillary temperature: 250 °C, capillary voltage: 30 V, tube lens: 150 V, and sheath gas flow rate: 10.

### 4.6. ESI-MS/MS and Database Search for Putative Compound Identification

ESI-MS/MS analyses of the most significant signals among different genotypes and treatments were performed by the same MS instrumentation described in the previous section, to intentionally fragment molecules into smaller parts for structure elucidation. Comparison of unknown compound MS/MS spectra against databases containing reference or predicted MS/MS spectra, is a widely used method for putative metabolite identification [47].

Each database employed in our study (Metlin [48], MassBank of North America—MoNA, https://mona.fiehnlab.ucdavis.edu (accessed on 21 June 2021), NIST-MS/MS library http://chemdata.nist.gov/mass-spc/msms-search/ (accessed on 21 June 2021) [49], and Saponins Mass Spectrometry Database [50]) has different mathematical/statistical approaches to rank the most probable compounds. The top ten compounds ranked were then manually inspected to identify the compound with highest number of fragment matches. In some cases, the compounds with higher scores (top rank) did not correspond to meaningful molecules, for instance they were high molecular weight compounds with multiple charges.

As a result of this search, lists of putative compound identification in positive and negative ion modes were created. The compounds were classified according to the confidence level as proposed by [51] (from level 0, unambiguous identification to level 4 unknown structure). Most of our findings reported in Table S2 are in level 3 (most likely structure).

## 5. Conclusions

Two major QTLs have been identified that have alleles that make large contributions to resistance to defoliating insects in soybean. The combination of these alleles is especially effective at producing resistance, and now has been shown to result in hundreds of metabolites that are upregulated in response to insect feeding.

How these QTLs lead to production of multiple metabolites is unknown. What is clear now is that searching for a single or few insecticidal compounds synthesized by these QTLs may not provide insights into the source of insect resistance; instead, these results reveal that defoliator resistance may be due to a cocktail of hundreds of metabolites.

From a crop perspective, such resistance is desirable, as it is difficult for defoliators to develop simultaneous resistance to hundreds of different compounds. The ability to obtain multiple resistance factors from just two loci is attractive from a breeding perspective due to its simplicity. As more of the genes involved and their associated compounds are identified, it may be possible to fine-tune the resistance in soybean, and use comparative genomics to create resistance in other legumes.

## Data Availability

Data is contained within the article or Supplementary Materials.

## Supplementary Materials

The following are available online at https://www.mdpi.com/article/10.3390/metabo11100710/s1. Figure S1: Principle component (PCA) and orthogonal PLS-PA analysis of soybean genotypes harboring insect resistance QTLs E, M, and EM compared to the introgressed susceptible parent Benning. Table S1: MS peaks from insect-resistant QTLs that were significantly different from insect-susceptible parent Benning. Table S2: Peak annotation and levels of confidence.
