# Supplementary material for: Metabolomics Differences of Glycine max QTLs Resistant to Soybean Looper
Source: Metabolites. 2021 Oct 19;11(10):710. doi: 10.3390/metabo11100710 (PMC8539317; doi:10.3390/metabo11100710)
Supplement: Supplementary file 1 [file metabolites-11-00710-s001.zip › Figure S1.pdf]

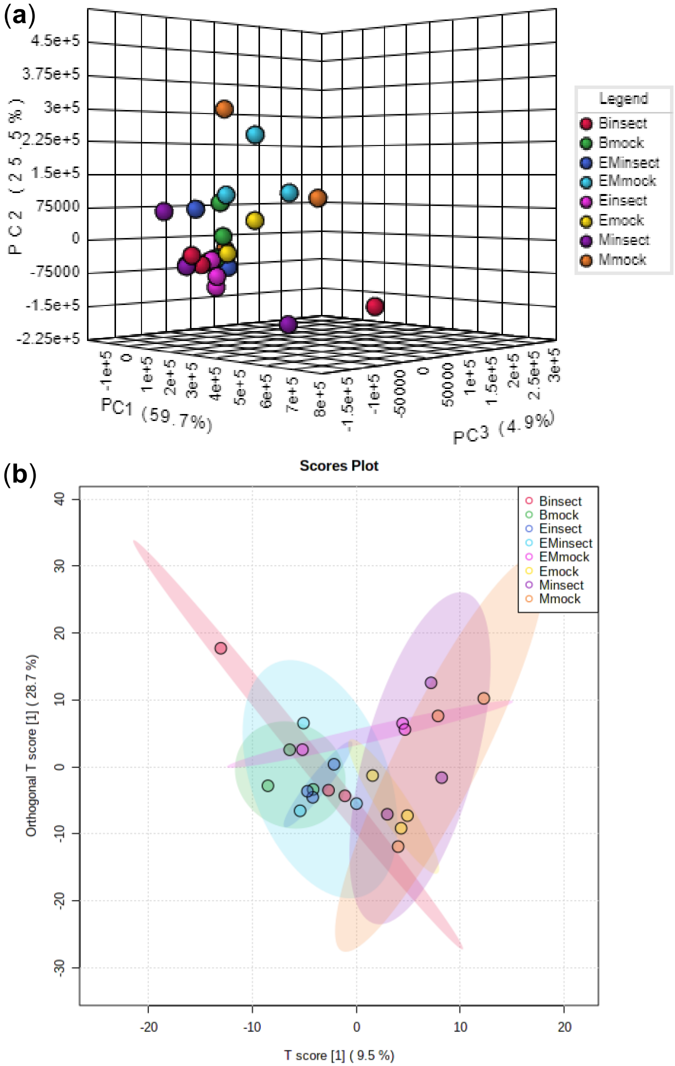

Figure S1. (a) Principle component (PCA) analysis of soybean genotypes harboring insect resistance QTLs E, M, and EM compared to the introgressed susceptible parent Benning. The analysis was based on 13,950 unique peaks identified by mass spectroscopy and was conducted using Metaboanalyst 5.0 (https://dev.metaboanalyst.ca/) with filtering based on relative standard deviation (RSD) with default parameters including no data transformation or scaling. **(b)** Orthogonal PLS-DA was conducted on the same data with filtering based on relative standard deviation (RSD) with default parameters including no data transformation or scaling. Ovals indicate 95% confidence intervals.
